# Construction of Gene Regulatory Networks Based on Spatial Multi-Omics Data and Application in Tumor-Boundary Analysis

**DOI:** 10.3390/genes16070821

**Published:** 2025-07-13

**Authors:** Yiwen Du, Kun Xu, Siwen Zhang, Lanming Chen, Zhenhao Liu, Lu Xie

**Affiliations:** 1 College of Food Science and Technology, Shanghai Ocean University, Shanghai 201306, China; duyiwen0926@163.com; 2 Shanghai-MOST Key Laboratory of Health and Disease Genomics, The Department of Genome and Bioinformatics, Shanghai Institute for Biomedical and Pharmaceutical Technologies, Fudan University, Shanghai 200237, China; xukun01102021@126.com (K.X.); zhangsiwen07@163.com (S.Z.); 3 Key Laboratory of Quality and Safety Risk Assessment for Aquatic Products on Storage and Preservation (Shanghai), China Ministry of Agriculture, College of Food Science, Shanghai Ocean University, Shanghai 201306, China; mchen@shou.edu.cn

**Keywords:** spatial transcriptomics, spatial proteomics, single-cell transcriptomics, spatial-resolved gene regulatory network, tumor boundary

## Abstract

Background/Objectives: Cell–cell communication (CCC) is a critical process within the tumor microenvironment, governing regulatory interactions between cancer cells and other cellular subpopulations. Aiming to improve the accuracy and completeness of intercellular gene-regulatory network inference, we constructed a novel spatial-resolved gene-regulatory network framework (spGRN). Methods: Firstly, the spatial multi-omics data of colorectal cancer (CRC) patients were analyzed. We precisely located the tumor boundaries and then systematically constructed the spGRN framework to study the network regulation. Subsequently, the key signaling molecules obtained by the spGRN were identified and further validated by the spatial-proteomics dataset. Results: Through the constructed spatial gene regulatory network, we found that in the communication with malignant cells, the highly expressed ligands *LIF* and *LGALS3BP* and receptors *IL6ST* and *ITGB1* in fibroblasts can promote tumor proliferation, and the highly expressed ligands *S100A8/S100A9* in plasma cells play an important role in regulating inflammatory responses. Further, validation of the key signaling molecules by the spatial-proteomics dataset highlighted the role of these genes in mediating the regulation of boundary-related cells. Furthermore, we applied the spGRN to publicly available single-cell and spatial-transcriptomics datasets from three other cancer types. The results demonstrate that ITGB1 and its target genes FOS/JUN were commonly expressed in all four cancer types, indicating their potential as pan-cancer therapeutic targets. Conclusion: the spGRN was proven to be a useful tool to select signal molecules as potential biomarkers or valuable therapeutic targets.

## 1. Introduction

The tumor microenvironment (TME) comprises diverse cell types whose coordinated interactions drive cancer progression [1]. Intercellular communication is fundamental to key biological processes, including metabolism, development, and immune responses [2,3]. Disruptions in these communication pathways can lead to disease. Thus, decoding intercellular communication networks is essential for understanding disease mechanisms and progression.

Single-cell RNA-seq enables high-resolution analysis of cellular heterogeneity but lacks spatial context, which is crucial given the localized nature of cell signaling [4,5]. Spatial transcriptomics overcomes this by mapping gene expression within tissue architecture, enabling improved analysis of cell–cell communication (CCC) [6]. Tools including CellPhoneDB [7], Giotto [8], and CellChat [9] could infer CCC networks with single-cell and spatial-transcriptomics data, yet they often neglect downstream transcriptional responses, limiting prediction accuracy [10]. An integrated pipeline is, thus, essential for constructing complete and reliable spatial gene regulatory networks.

In previous work, we developed the cIGRN, an integrative pipeline for constructing scRNA-seq-based intercellular gene regulatory networks, validated with spatial transcriptomics [11,12]. To leverage the unique features of spatial multi-omics data and optimize key signaling molecule screening, here, in this work, we expanded this process into a new spatial-resolved gene-regulatory network pipeline (spGRN) (https://github.com/duyiwen026/spGRN, accessed on 11 July 2025). The spGRN fully retains spatial information by integrating multiple intercellular communication tools, and it encompasses not only ligand–receptor interactions but also systematically incorporates downstream transcription factors and target gene regulation, significantly enhancing the interpretation of intercellular communications [13,14,15]. Given that the tumor boundary represents the interface between the tumor and adjacent normal tissue, this area is very active in tumor invasion and metastasis [16]. We locate the tumor boundaries and study the network regulation. There is increasing evidence that fibroblasts are involved in direct communication, affecting the entire tissue microenvironment and influencing disease outcomes [17,18]. Meanwhile, plasma cells play a significant role in creating the chronic inflammatory milieu that is characteristic of CRC, thereby influencing both tumor promotion and anti-tumor immunity [19,20]. Therefore, in addition to the immune cell subsets with strong interactions in solid tumors, we also conducted an in-depth analysis of the regulatory network between fibroblasts and malignant cells.

Proteins, as the primary effectors of cellular function, directly participate in biological processes and serve as the core molecules through which regulatory networks exert their ultimate effects [21,22], thereby providing a more accurate representation of the functional state of genes [23]. Transcriptomics and proteomics inherently represent interconnected upstream/downstream molecular layers [24]. Their integration exploits complementary features to comprehensively reconstruct biological networks and uncover multi-level tumor progression mechanisms [25]. Therefore, we combined key signaling molecules from regulatory network analysis with spatial-proteomics data to precisely delineate the molecular landscape of tumor-boundary regions.

In this study, we analyzed the spatial multi-omics data of colorectal cancer patients. Furthermore, we applied a spGRN to publicly available single-cell and spatial-transcriptomics datasets from three other cancer types. This study provides an effective method for screening key regulatory signals in the tumor microenvironment, and the selected signaling molecules identified by the spGRN might be potential biomarkers for mechanistic studies or pan-cancer therapeutic targets.

## 2. Materials and Methods

### 2.1. Single-Cell and Spatial-Transcriptomics Data Collection

Publicly available single-cell and spatial-transcriptomics data were collected for this study. The scRNA-seq datasets for colorectal cancer (CRC) were obtained from GEO under accession numbers GSE161277 [26], GSE231559 [27], and GSE231993 [28]. The scRNA-seq datasets of the CRC samples obtained from GSE205506 [29] were treated as an independent validation cohort. Spatial-transcriptomics (ST) datasets for colorectal cancer were sourced from the scCRLM Atlas (http://www.cancerdiversity.asia/scCRLM/, accessed on 22 November 2023) [30] and 10× Genomics (Pleasanton, CA, USA). Expanded application analyses were performed on three other cancer types. For breast cancer, scRNA-seq datasets were obtained from GSE161529 [31] and GSE176078 [32]. The scRNA-seq datasets for ovarian cancer were acquired from GSE184880 [33]. The scRNA-seq dataset for lung cancer was downloaded from CodeOcean (https://codeocean.com/capsule/8321305/tree/v1, accessed on 7 March 2024) [34]. The spatial-transcriptome data for the three cancer types were obtained from the 10× Genomics website. Detailed information can be found in the data availability statement section.

### 2.2. Dimension Reduction and Clustering Analysis of Single-Cell Transcriptome Data

Single-cell transcriptomics data analysis was performed with Seurat (v4.3.0) [35]. Cells with a mitochondrial gene content >20%, unique molecular identifiers (UMIs) <200 or >60,000, and detected genes <200 were excluded during quality control. Data normalization and scaling were performed using the NormalizeData and ScaleData functions, respectively. Principal component analysis (PCA) was conducted on highly variable genes, followed by the construction of a shared nearest neighbor graph (FindNeighbors) and unsupervised clustering analysis (FindClusters). Uniform manifold approximation and projection (UMAP) was used for dimensionality reduction and visualization. Batch effects were corrected using Harmony (v1.1.0) [36]. Marker genes were identified using FindAllMarkers, and cell types were annotated using SingleR [37] (v2.2.0), leveraging annotations from the CellMarker database [38] and curated marker genes.

### 2.3. Identification of Malignant Cells from scRNA-Seq Data

To distinguish malignant from normal epithelial cells, we calculated somatic large-scale chromosome copy number variation (CNV) scores using inferCNV (v1.16.0). A raw counts matrix, annotation file, and gene/chromosome position file were prepared. Epithelial cells from normal samples served as the reference group, while tumor epithelial cells composed the observation group. Cells with significantly elevated CNV scores compared with the reference group were classified as malignant.

### 2.4. Spatial-Transcriptomics Data Analysis

Spatial-transcriptomics data were generated using the 10× Genomics Visium platform following the standard protocol and processed with Space Ranger v1.1. Gene-spot matrices were analyzed in Seurat, filtering for spots with ≥200 detected genes and genes expressed in ≥3 spots with ≥10 counts. Cell-type distributions from the scRNA-seq were projected onto the ST data using AddModuleScore, estimating the cell-type proportions per spot. SpatialFeaturePlot was used to visualize the spatial expression patterns across tissue sections.

### 2.5. Spatial Cell Communication Strength Analysis

Cell–cell communication was analyzed using CellChat v2, with CellChatDB.human as the reference [39]. To emphasize local interactions, distant communications were excluded by setting distance.use = FALSE. Communication probabilities for each signaling pathway were computed using computeCommunProbPathway. Integrated communication among cell types was summarized with aggregateNet and visualized using netVisual_heatmap.

### 2.6. Definition of Tumor-Boundary-Based Spatial-Transcriptome Data

STInferCNV and STCNVScore in Cottrazm [40] were used to define the highest CNV score as the core tumor spot. The BoundaryDefine function was used to determine the malignant, tumor-boundary, and non-malignant regions. Finally, the BoundaryPlot function was employed to visualize the annotation results of the regions.

### 2.7. Flow for Constructing Spatial Gene Regulatory Networks

To construct a spatially resolved gene regulatory network (spGRN) within the tumor microenvironment, we applied a multi-step integrative pipeline that systematically combines spatial-transcriptomics data with cell–cell communication and gene-regulatory network inference tools. First, SpaTalk [41] was used to perform a spot-level analysis of spatially resolved cell–cell communication. This step enabled the identification of spatially proximal cell types and prediction of active ligand–receptor interactions, with malignant cells designated as the sender population to investigate their influence on the surrounding microenvironment. Next, stLearn [42] was employed to refine ligand–receptor pair identification by integrating spatial coordinates with gene expression and histological features, thereby enhancing the spatial specificity of predicted interactions. All analyses were performed using human-specific default reference databases, as outlined below. We applied stringent filtering criteria, retaining only the top 200 ligand–receptor pairs with adjusted *p*-values < 0.05 (pval_adj_cutoff = 0.05 and n_pairs = 200) to ensure high-confidence interactions. To further dissect the downstream regulatory effects within malignant cells, we used pySCENIC (v0.11.2) [43]. pySCENIC identifies transcription factors (TFs) and their target genes and prunes indirect interactions through motif enrichment analysis, yielding high-confidence regulons within the malignant cell population. Finally, differentially expressed genes (DEGs) were identified by the FindMarkers function in Seurat. The pruned gene regulatory networks were, ultimately, integrated as cell-interaction-based microenvironmental gene regulatory networks, serving as the final version for subsequent analyses. The cIGRN strategy, previously published by our group, was updated in this study as well. The method of the spatial regulation network is currently embedded in the pipeline spGRN constructed in this work.

### 2.8. Spatial-Proteomic Profiling Analysis

Spatial-proteomics data for colorectal cancer (CRC) were obtained from the iProX database (project number: IPX0007407000) [44]. Raw mass spectrometry (MS) data were processed using MaxQuant (v2.4.7.0) following standard workflows for MS-based proteomics analysis. Subsequent statistical analysis was conducted with Perseus (v2.6.0), where contaminant proteins, reverse hits, and peptides with non-unique sequences (peptide count >1) were removed to ensure data quality and specificity. Differential protein expression analysis was performed using the R package DEP (v1.22.0), enabling robust identification of significantly altered proteins across spatial regions. To further explore the biological relevance of these proteins, a protein–protein interaction (PPI) network was constructed using high-confidence interactions from the STRING database (https://cn.string-db.org, accessed on 18 march 2024) [34]. This network facilitated the identification of key functional modules and biological pathways associated with spatial-proteomic alterations in CRC. Finally, Cytoscape (v3.10.3) was used to plot the PPI network diagram.

### 2.9. Enrichment Analysis and Survival Analysis

Survival analyses of target genes were conducted using GEPIA2 [45] and Kaplan–Meier plotter [46]. For enrichment analysis, gene IDs were converted using AnnotationDbi and clusterProfiler, followed by GO and KEGG enrichment analyses. Pathways from the MSigDB hallmark gene sets with an adjusted *p*-value < 0.05 were considered statistically significant.

## 3. Results

### 3.1. Single-Cell and Spatial-Transcriptome Profiles of Colorectal Cancer

**Figure 1:**
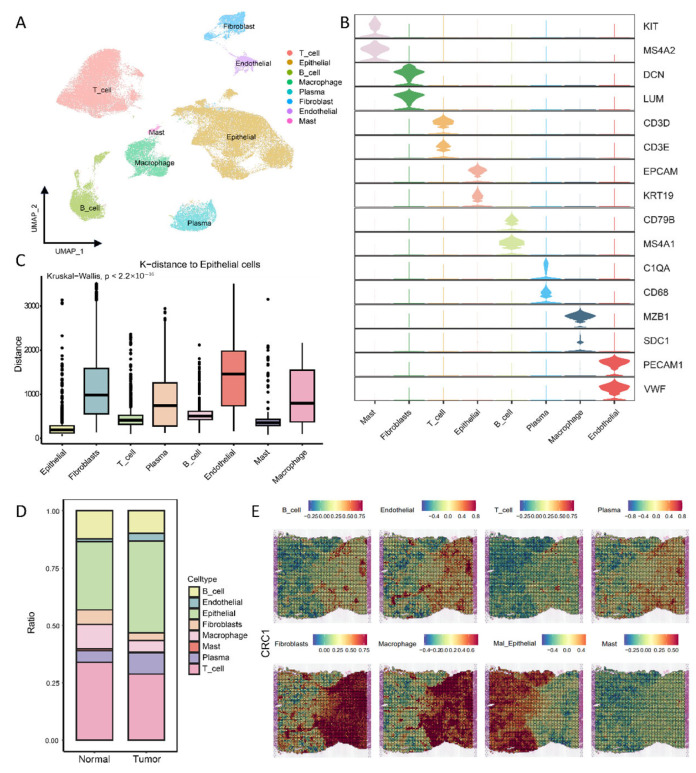
Single-cell and spatial-transcriptomic profiles of colorectal cancer: (**A**) UMAP plot showing cell populations in colorectal cancer; (**B**) violin plots with markers for eight cell types showing the gene expression distributions; (**C**) CellTrek-generated boxplots revealing the intercellular distances between epithelial and other cell types; (**D**) proportions of cell types in normal vs. tumor groups highlighting the compositional differences; (**E**) unbiased clustering of spatial-transcriptomics (ST) points showing the spatial localization of the major cell types.

To comprehensively characterize the single-cell landscape of CRC, we integrated scRNA-seq data of 105,316 cells from 10 normal colorectal samples and 18 colorectal cancer patient samples for analysis. All cells were classified into the following eight cell types based on their marker genes: mast cells (MS4A2, KIT), fibroblast cells (LUM, DCN), T cells (CD3D, CD3E), epithelial cells (ECAM1, KRT19), B cells (CD79B, MS4A1), plasma cells (MZB1, SDC1), macrophage cells (CD68, C1QA), and endothelial cells (PCAM1, VWF) (Figure 1A,B). It should be noted that with the progression of CRC, a higher proportion of epithelial cells was observed in the tumor samples (Figure 1D and Figure S1A). Since CRC originates from colorectal epithelial cells, primarily driven by DNA copy number variations (CNVs), we performed CNV analysis on the single-cell transcriptome data to identify malignant cells within the dataset (Figure S1B). In addition, in order to clarify the tumor microenvironment of colorectal cancer, we collected six colorectal cancer spatial-transcriptome samples (CRC1-CRC6). The spatial distances among cells were inferred (Figure 1C and Figure S1C), and the spatial distribution of the cells was estimated (Figure 1E and Figure S2). These results indicate that immune cells are mainly concentrated around epithelial cells and fibroblasts. This spatial distribution strongly suggests that there are complex intercellular interactions among these three different cell types.

### 3.2. Tumor-Boundary Cell Interactions

**Figure 2:**
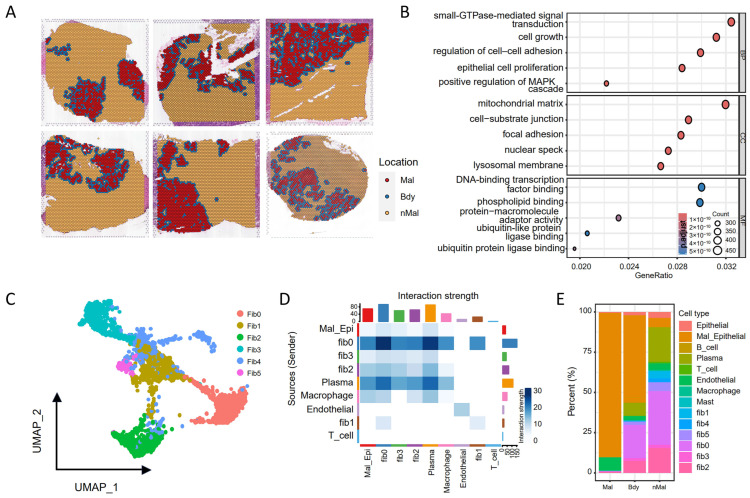
Tumor microenvironment in colorectal cancer spatial transcriptomics: (**A**) spots from six CRC samples annotated as malignant (Mal, red), boundary (Bdy, blue), and non-malignant (nMal, orange) regions; (**B**) GO enrichment analysis of boundary spots (Bdy); (**C**) UMAP plot of fibroblast subpopulations; (**D**) heatmap of the intercellular interaction intensity; (**E**) cell-type proportions across malignant, boundary, and non-malignant regions.

Tumor tissues have complex spatial architecture, and the rigorous analysis of the spatial microenvironment is critical to understanding tumorigenesis, progression mechanisms, and discovery of therapeutic targets [47]. To address this, spatial-transcriptomics data of six colorectal cancer samples were analyzed (CRC1-CRC6). Each spatial slice was classified into the following three regions: malignant (Mal), boundary (Bdy), and non-malignant (nMal) (Figure 2A) [36]. Differential expression analysis was performed between Bdy and Mal regions to identify differentially expressed genes (DEGs) (adjusted *p*-value < 0.05). Functional enrichment analysis for the DEGs reveals that the Mal region was primarily enriched for biological processes associated with cellular respiration, oxidative phosphorylation, and other common tumor-related bioactivities (Figure S3). In contrast, the Bdy region exhibited significant enrichment in biological processes associated with cell growth, proliferation, and signal transduction. This finding implies that this area is highly active in tumor cell invasion and intercellular interactions (Figure 2B). Thus, the spatial heterogeneity, composition, and cellular interactions within the tumor boundary might play critical roles in cancer progression.

To further investigate the cellular composition and intercellular interactions at the tumor boundary, we performed a detailed cell proportion analysis by integrating single-cell transcriptomic data. Given the pronounced heterogeneity of fibroblasts, we conducted a more refined clustering analysis, identifying six distinct fibroblast subpopulations (Figure 2C). Cell interaction analysis identified fib0, fib2, and plasma cells as having the strongest boundary interactions with malignant cells (Figure 2D); this echoes the significant changes in their proportions (Figure 2E). These findings suggest that fibroblasts and plasma cells engage in cellular interactions and network regulation within the tumor microenvironment, potentially influencing subpopulation dynamics and malignant cell proliferation. To further elucidate the signaling molecules involved in these boundary-specific interactions, we plan to construct a spatial cellular regulatory network for deeper mechanistic insights.

### 3.3. Spatial-Resolved Gene-Regulatory Network Construction at the Cell Tumor Boundary

**Figure 3:**
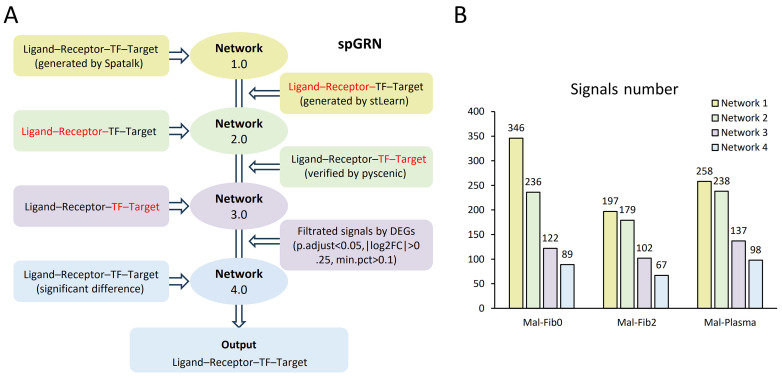
Construction of the spatially resolved gene regulatory network at tumor boundaries: (**A**) workflow for the spGRN’s construction; (**B**) changes in signal quantities during the spGRN’s construction.

In order to understand the complex and dynamic mutual regulatory relationships among subpopulations at spatial boundaries, we constructed a spatial-based cell regulatory network. First, we used Spatalk to reveal spatially resolved intercellular communication from single-cell and spot-based ST data, obtain spatial signal transduction networks, and construct ligand–receptor–TF–target connections (Network 1.0); then, stLearn was used with the spatial information and gene expression profiles to determine tissues with high ligand–receptor interaction activity and retain overlapping ligand–receptor pairs and their downstream signals (Network 2.0); then, pySCENIC was used to verify the transcription factors in cells and prune the gene regulatory networks (Network 3.0). Finally, differentially expressed genes (DEGs) were used for screening to filter important signals for Network 3.0 (Network 4.0) (Figure 3A). After multiple screenings of the regulatory network, the signal molecules were reduced, which means that overlapping common filters derived from the analysis of multiple communication tools can reduce false-positive results using a single tool. For example, after the four-layer network analysis of plasma cells and malignant cells by the spGRN, the signal decreased from 258 to 98 (Figure 3B). The advantage of this process is the accuracy of the screening signal molecules. Idle data usually have high noise and heterogeneity, and multiple spatial tools were employed in the spGRN pipeline to reduce false positives at each node and improve the accuracy and reliability of the results through multi-omics-integrated analysis. In this study, we focused on the construction of a spatial-resolved gene regulatory network between plasma cells/fibroblasts and malignant cells at the tumor boundary based on previous findings.

### 3.4. spGRN Analysis of Plasma/Fibroblast Cells and Malignant Cells at the Spatial Boundary

**Figure 4:**
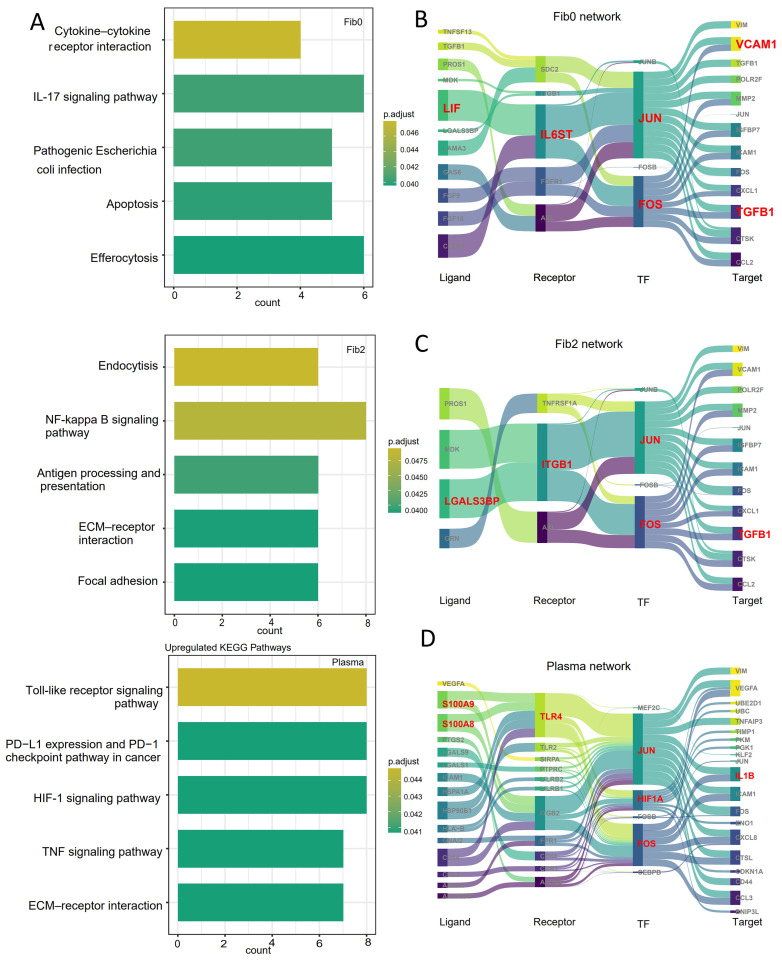
Spatially resolved gene regulatory networks from the spGRN: (**A**) KEGG pathways enriched in fibroblast subsets (fib0 and fib2) and plasma cells at tumor boundaries; (**B**–**D**) spatially resolved gene regulatory networks of malignant cells interacting with fib0 (**B**), fib2 (**C**), and plasma cells (**D**).

Based on the above findings, we focused on fibroblast subsets (fib0 and fib2) and plasma cells to explore the molecular mechanisms by which these cell populations may mediate tumor cell alterations at the tumor boundary. First, we performed KEGG pathway enrichment analysis on these three cell subsets. The results show that fib0 was primarily enriched in the cytokine–cytokine receptor interaction and IL-17 signaling pathway, while fib2 was enriched in the NF-kappa B signaling pathway and focal adhesion pathway. In contrast, plasma cells were significantly enriched in the HIF-1 signaling pathway and TNF signaling pathway (Figure 4A). To further identify key molecules involved in tumor microenvironment remodeling within these pathways, spatial-resolved gene regulatory networks were constructed with the spGRN.

In the interaction network between fib0 and malignant cells, we identified a key axis involving LIF/CLCF1–IL6ST–JUN/FOS–ICAM1. Notably, IL6ST plays crucial roles in maintaining immune homeostasis, inflammatory responses, and metabolic regulation within the tumor microenvironment [48]. Within the IL-17 signaling pathway, the interaction between IL-17 and IL-6 triggers inflammatory responses, consistent with previous findings that IL-6 activity is essential downstream of IL-17 signaling (Figure 4B) [49]. In the interaction network between fib2 and malignant cells, we identified a key axis involving LGALS3BP–ITGB1–JUN/FOS–TGFβ1 (Figure 4C), which is primarily associated with tumor migration, cell adhesion, and angiogenesis. A positive correlation was observed between LGALS3BP and TGFβ1 levels, with LGALS3BP directly binding to integrins and facilitating the release of TGF-β1 [50]. The released TGF-β1 subsequently activates the JUN transcription factor, reinforcing a positive feedback loop that promotes tumor progression.

Similarly, we constructed a gene regulatory network between plasma cells and malignant cells. Through the spGRN analysis, we identified 98 signaling molecules involved in this network. Combining these findings with our enrichment analysis, we identified the S100A8/S100A9–TLR4–HIF1A–IL1B axis as a key mediator of plasma cell–malignant cell interactions (Figure 4D). TLR4 is a key receptor for S100A8 in regulating macrophage function. When S100A8 and S100A9 are secreted into the extracellular space, they act as pro-inflammatory danger signals and have been shown to play a pivotal role in tumorigenesis by influencing inflammation, proliferation, invasion, and metastasis of tumor cells [51]. In the HIF-1 signaling pathway, hypoxia-inducible factor HIF1A is produced under hypoxic conditions, which drives the secretion of IL1B to enhance tumor cell proliferation, migration, and invasion [52,53]. These findings suggest that the identified signaling molecules could serve as potential prognostic markers and therapeutic targets in cancer.

### 3.5. Independent Validation of Main Signaling Molecules in CRC

**Figure 5:**
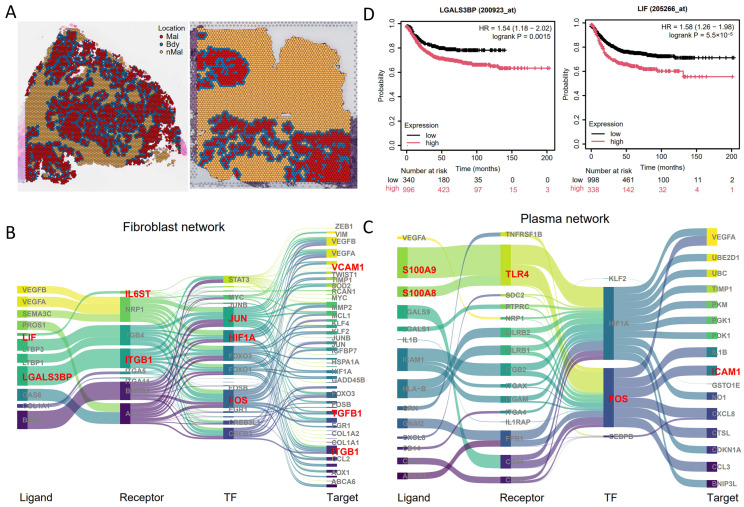
Spatially resolved gene regulatory networks validated using an independent CRC dataset: (**A**) annotated spots in the validation dataset; (**B**–**C**) spatially resolved gene regulatory networks of malignant cells interacting with fibroblasts (**B**) and plasma cells (**C**); (**D**) Kaplan–Meier estimates of overall survival (OS) for LGALS3BP and LIF from the TCGA CRC RNA-seq dataset.

To validate our gene-regulatory network (GRN) screening and key signaling molecules, we utilized an independent validation cohort of colorectal cancer scRNA-seq and spatial transcriptomics (CRC7, CRC8) (Figure 5A, Figures S4 and S5), processed identically to the experimental cohort. Subsequent spGRN-based regulatory network analysis further supports the robustness of our findings, as several key signaling axes identified in the experimental cohort were also detected in the validation cohort. For instance, within the fibroblast–malignant cell interaction network, the LIF/CLCF1–IL6ST–JUN/FOS–ICAM1 axis and the LGALS3BP–ITGB1–JUN/FOS–TGFB1 axis were consistently observed (Figure 5B). Similarly, within the plasma cell–malignant cell interaction network, the S100A8/S100A9–TLR4–HIF1A–IL1B axis was identified again (Figure 5C), reinforcing the validity of the spGRN method. To further evaluate the clinical relevance of the identified key signaling molecules both in the experimental and validation groups, we conducted a prognostic analysis of the genes LGALS3BP and LIF. We found that the high expression of these two genes is significantly associated with a poorer survival outcome in colorectal cancer patients (Figure 5D). These results emphasize the potential of these signaling molecules as biomarkers and therapeutic targets.

### 3.6. Validation of the spGRN Results Using Spatial-Proteomics Data

Given the hierarchical relationship between transcriptomics and proteomics in biological regulation, their integrative analysis leverages complementary insights to achieve a more comprehensive understanding of molecular mechanisms.

**Figure 6:**
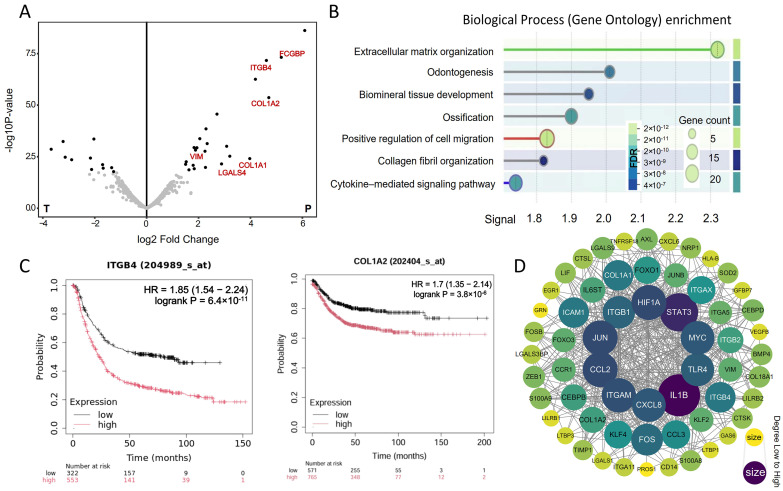
Key proteins validated from the spatial proteomics: (**A**) differentially expressed proteins in colorectal cancer and adjacent tissues; (**B**) GO biological process enrichment analysis for the top DEPs; (**C**) KM estimate of the overall survival (OS) for *ITGB4* from the TCGA CRC RNA-seq dataset; (**D**) PPI network of the DEPs.

The results show that, compared with the tumor area, the protein abundance in the tumor-adjacent tissues is higher, and there is a significant difference in the protein expression profile (Figure 6A). To explore the functional implications of these differentially expressed proteins, we performed an enrichment analysis of the Gene Ontology (GO) biological process (BP) for the highly expressed proteins in the cancer-adjacent tissues (Figure 6B). The analysis highlighted a significant enrichment of tumor-boundary-associated biological processes, predominantly involving cell migration and signal transduction. These findings underscore the crucial role of tumor-adjacent tissues in regulating the tumor microenvironment. Further examination of the DEPs revealed that several signaling molecules previously identified at the tumor boundary, including ITGB4, COL1A2, and VIM, were significantly upregulated in the cancer-adjacent tissue (*p*.adjust < 0.05). The observation that the gene expression profiles in the tumor-boundary regions closely resembled those of the tumor-adjacent tissues may reflect the dynamic infiltrative nature of the tumor microenvironment. Genes such as COL1A2, VIM, and TGFB1/TGFB4, which are associated with tumor cell invasiveness and tissue remodeling, were not only expressed in infiltrative tumor cells but also broadly present in the surrounding peritumoral tissues influenced by the tumor milieu (Figure 6C). Subsequently, we performed a PPI analysis on the differentially expressed proteins with high expression and the key signaling molecules identified in the boundary regulatory network (Figure 6D). The results demonstrate significant interactions between these proteins and the ITGB family, forming tightly connected nodes within multiple signaling pathways, further confirming the ITGB integrin family as key regulators of colorectal cancer.

### 3.7. Reproducible Regulatory Networks Identified in Pan-Cancer Using the spGRN

**Figure 7:**
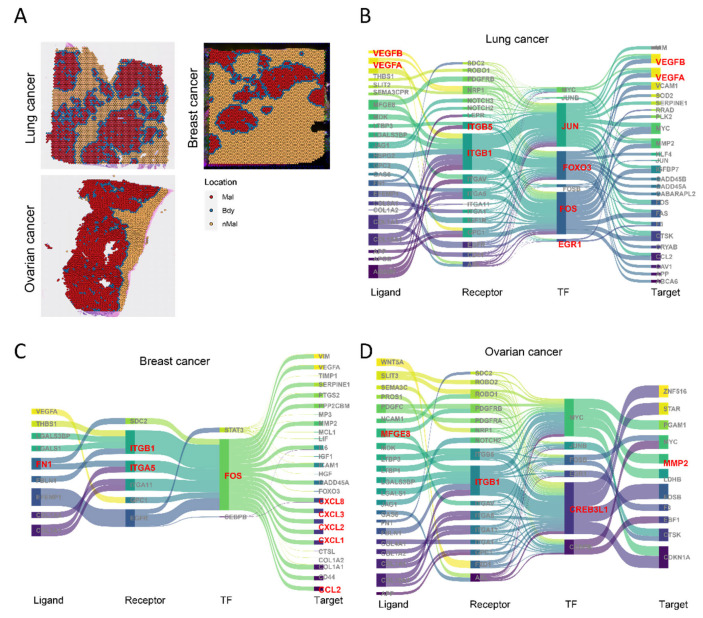
Spatially resolved gene regulatory networks across cancer types: (**A**) spots from lung, breast, and ovarian cancer samples, annotated; (**B**–**D**) spatially resolved gene regulatory networks of malignant cells interacting with fibroblasts in lung (**B**), breast (**C**), and ovarian (**D**) cancers.

To extend our findings, we applied the spGRN framework to a pan-cancer analysis integrating single-cell and spatial-transcriptomic datasets from lung (122,148 cells), breast (96,794 cells), and ovarian (85,698 cells) cancers (Figure 7A and Figure S6–S8). Focusing on the critical role of fibroblasts in the tumor microenvironment, we systematically constructed fibroblast–malignant cell regulatory networks across these cancers. This revealed key signaling molecules and pathways associated with progression, including angiogenesis, proliferation, survival, immune evasion, and inflammation, for example, VEGFA/VEGFB [54,55], FOXO3 [56], and PDGFRB [57] in lung cancer angiogenesis (Figure 7B); CCL2 [58] and CXCL12 [59] chemokines modulating CAF activity in breast cancer invasion/metastasis (Figure 7C); and MFGE8 [60] and MYC [61] promoting survival, EMT, and phagocytosis in ovarian cancer (Figure 7D). Notably, conserved overexpression of JUN/FOS (AP-1 complex) [62] and ITGB1 [63] was observed across all three cancer types and colorectal cancer (Figure 7B–D), underscoring their roles as shared molecular drivers. JUN/FOS regulate proliferation, differentiation, apoptosis, and inflammation, with aberrant activation promoting tumor growth and invasion, while ITGB1 critically mediates cell adhesion, migration, and signaling. Further investigation of these pathways will elucidate their core pan-cancer functions and advance therapeutic strategies.

This systematic analysis not only verified the versatility of the spGRN method across different cancer types but also revealed the high complexity and dynamics of tumor-boundary regions. These results provide strong support for further exploring the diversity and spatial regulation mechanisms of the tumor microenvironment and, at the same time, establish the reliability of the spGRN in the spatial regulation network.

## 4. Discussion

In this study, we conducted a comprehensive analysis of the tumor microenvironment (TME) at tumor boundaries by integrating scRNA-seq and spatial-transcriptomics (ST) datasets with spatial-resolved gene regulatory networks (spGRN). At the same time, spatial mass spectrometry data were introduced for verification. This approach enabled us to uncover spatially resolved intercellular gene regulatory networks and identify plausible signaling pathways at the spatial level. By combining these methodologies, our spatial multi-omics-based analysis mitigates the limitations of individual approaches [25].

Intercellular communication influences cell phenotype and function. Currently, most intercellular communication is ligand–receptor based, and analysis methods for different intercellular communication tools result in different scores that are difficult to compare and evaluate, making it challenging to validate the results [64,65]. To better capture the dynamics of these interactions, we developed and refined a pipeline for constructing spatial-resolved gene regulatory networks (spGRNs). While scRNA-seq captures ligand–receptor gene expression, which is critical for CCC detection, it lacks spatial context and ignores key constraints of potential interactions. The spGRNs use multiple CCC tools for integration, minimize false positives, and integrate spatial localization, retaining the advantages of ST data. Unlike traditional CCC methods that focus primarily on intercellular signaling, spGRNs incorporate downstream cellular responses. By analyzing the ligand–receptor–transcription factor–target gene cascade, spGRNs provide a more comprehensive view of intercellular communication, enhancing its completeness and biological relevance.

Malignant cells at tumor boundaries are highly invasive and play a pivotal role in reshaping the tumor microenvironment [66]. Spatial multi-omics data analysis of colorectal cancer (CRC) revealed strong interactions among fibroblasts, plasma cells, and malignant cells at the boundary. As regions transition from non-malignant to malignant, immune and fibroblast proportions decline, while malignant cell abundance increases, underscoring their role in cell–cell communication. Using spGRNs, we identified key boundary-specific regulatory axes, including LIF/CLCF1-IL6ST-JUN/FOS-ICAM1 and LGALS3BP-ITGB1-JUN/FOS between malignant cells and fibroblasts, as well as S100A8/S100A9-TLR4-HIF1A-IL1B between malignant cells and plasma cells. The key regulatory molecules identified in the CRC validation datasets exhibit similar expression patterns and are associated with poor prognosis, confirming the reliability and consistency of the spGRN-based analysis.

In addition, we also collated the expression data of spatial proteins at the spatial level. The data from taking the spatial positioning as the tumor site and the normal part of the tumor boundary were analyzed. By integrating the key signaling molecules from the regulatory network, we sought to gain deeper insights into the functional patterns of critical genes across different regulatory levels. Through protein–protein interaction (PPI) analysis, we further confirmed the central role of the ITGB family within the regulatory network, revealing that it may mediate dynamic changes in the tumor microenvironment by synergistically regulating other signaling molecules. Finally, we applied the spGRN pipeline to lung cancer, breast cancer, and ovarian cancer, systematically constructing and analyzing the regulatory networks between fibroblasts and malignant cells to explore cross-cancer commonalities in their regulatory patterns. Interestingly, we identified the JUN/FOS and ITGB families as key regulators in tumor biology. Therefore, further investigation into the signaling pathways mediated by JUN/FOS and ITGB may offer new directions for developing pan-cancer precision therapies.

In this study, we integrated single-cell transcriptomics, spatial transcriptomics, and spatial proteomics to construct a multi-dimensional view of the tumor microenvironment. Meanwhile, by synergistically leveraging single-cell and spatial multi-omics perturbation screening, we aim to provide more comprehensive insight into cell states and intercellular communication within spatial contexts, thereby bridging the gap between molecular profiles and tissue-level organization. With the continuous advancement of spatial technology, cutting-edge tools have become increasingly powerful in exploring organizational structures. Our upcoming research will apply advanced spatial proteome image analysis techniques to gain a deeper understanding of the spatial location distribution of proteins. Notably, the selected signal molecules hold great promise as potential biomarkers for mechanism research or valuable therapeutic targets. Future studies could primarily concentrate on delving into the specific functions and application prospects of these molecules in the realm of cancer treatment. The spGRN described in this work may potentially shed light on developing new approaches for cancer therapy.

## Data Availability

The single-cell and spatial-transcriptomics datasets utilized in this study were retrieved from publicly available databases. Details are as listed below: Single-cell transcriptomics datasets for colorectal cancer (GSE161277, GSE231559, GSE231993, and GSE205506), breast cancer (GSE161529 and GSE176078), and ovarian cancer (GSE184880) were obtained from Gene Expression Omnibus. The single-cell transcriptomics datasets for lung cancer were acquired from CodeOcean (https://codeocean.com/capsule/8321305/tree/v1, accessed on 7 March 2024). Spatial-transcriptomics (ST) datasets for colorectal cancer were sourced from the scCRLM Atlas (CRC1-6: http://www.cancerdiversity.asia/scCRLM/, accessed on 22 November 2023). The ST dataset, as the independent CRC validation group, were obtained from the 10× Genomic website (CRC7: https://www.10xgenomics.com/datasets/human-colorectal-cancer-11-mm-capture-area-ffpe-2-standard, accessed on 22 November 2023) and Genome Sequence Archive (GSA) (CRC8: HRA000979). The spatial-transcriptome data for the other three cancer types, including lung cancer (https://www.10xgenomics.com/datasets/human-lung-cancer-ffpe-2-standard, accessed on 10 September 2024), breast cancer (https://www.10xgenomics.com/datasets/invasive-ductal-carcinoma-stained-with-fluorescent-cd-3-antibody-1-standard-1-2-0, accessed on 10 September 2024) and ovarian cancer (https://www.10xgenomics.com/datasets/human-ovarian-cancer-11-mm-capture-area-ffpe-2-standard, accessed on 10 September 2024), were obtained from the 10× Genomics website.

## Supplementary Materials

The following supporting information can be downloaded at: https://www.mdpi.com/article/10.3390/genes16070821/s1, Figure S1: Cell-type proportions, epithelial cell copy number changes, and cellular spatial distance analysis: (A) proportions of eight cell types in 28 colorectal cancer samples; (B) heatmap of CNV profiles across epithelial cell clusters, where red/blue shades indicate elevated/reduced CNVs compared with normal epithelial cells; (C) CellTrek-assessed boxplot of intercellular distances between fibroblasts and other cell types. Figure S2: Spatial-transcriptomic profiles for colorectal cancer. Figure S3: GO enrichment analysis of malignant (Mal) regions. Figure S4: Malignant region enrichment and independent CRC dataset profiles: (A) violin plots with markers for eight cell types; (B) UMAP plot showing distinct cell-type populations; (C) heatmaps of intercellular interaction number (left) and intensity (right). Figure S5: Spatial distance analysis in two independent CRC datasets: (A) unbiased clustering of ST points depicts spatial localization of major cell types, with red/blue indicating high/low group-specific expression (CRC7-8); (B) CellTrek-assessed boxplot of intercellular distances between epithelial and other cell types; (C) spatial-transcriptome cell colocalization analysis. Figure S6: Single-cell and spatial-transcriptomic profiles for lung cancer: (A) violin plots with markers for seven cell types; (B) UMAP plot showing distinct cell-type populations; (C) heatmap of CNV profiles across epithelial cell clusters, with red/blue shades indicating elevated/reduced CNVs compared with normal epithelial cells; (D) unbiased clustering of ST points revealing spatial localization of the major cell types; (E) Kaplan–Meier estimates of overall survival (OS) for *VEGFA* from the TCGA lung cancer RNA-seq dataset. Figure S7: Single-cell and spatial-transcriptomic profiles for breast cancer: (A) violin plots with markers for eight cell types; (B) UMAP plot showing distinct cell-type populations; (C) heatmap of CNV profiles across epithelial cell clusters, with red/blue shades indicating elevated/reduced CNVs compared with normal epithelial cells; (D) unbiased clustering of ST points revealing spatial localization of the major cell types; (E) Kaplan–Meier estimates of overall survival (OS) for *FN1* from the TCGA breast cancer RNA-seq dataset. Figure S8: Single-cell and spatial-transcriptomic profiles for ovarian cancer: (A) violin plots with markers for six cell types; (B) UMAP plot showing distinct cell-type populations; (C) heatmap of CNV profiles across epithelial cell clusters, with red/blue shades indicating elevated/reduced CNVs compared with normal epithelial cells; (D) unbiased clustering of ST points revealing spatial localization of the major cell types; (E) Kaplan–Meier estimates of overall survival (OS) for *MMP2* from the TCGA ovarian cancer RNA-seq dataset. Figure S9: Survival analysis of the key molecules: (A-B) Kaplan–Meier estimate of overall survival (OS) for *ITGB1* (A) and *FOS* (B) from the TCGA CRC RNA-seq dataset.

## References

[1] Armingol E., Officer A., Harismendy O., Lewis N.E. (2021). Deciphering cell-cell interactions and communication from gene expression. Nat. Rev. Genet..

[2] Su J., Song Y., Zhu Z., Huang X., Fan J., Qiao J., Mao F. (2024). Cell-cell communication: New insights and clinical implications. Signal Transduct. Target. Ther..

[3] Schürch C.M., Bhate S.S., Barlow G.L., Phillips D.J., Noti L., Zlobec I., Chu P., Black S., Demeter J., McIlwain D.R. (2020). Coordinated Cellular Neighborhoods Orchestrate Antitumoral Immunity at the Colorectal Cancer Invasive Front. Cell.

[4] Wang S., Sun S.T., Zhang X.Y., Ding H.R., Yuan Y., He J.J., Wang M.S., Yang B., Li Y.B. (2023). The Evolution of Single-Cell RNA Sequencing Technology and Application: Progress and Perspectives. Int. J. Mol. Sci..

[5] Liao J., Lu X., Shao X., Zhu L., Fan X. (2021). Uncovering an Organ’s Molecular Architecture at Single-Cell Resolution by Spatially Resolved Transcriptomics. Trends Biotechnol..

[6] Longo S.K., Guo M.G., Ji A.L., Khavari P.A. (2021). Integrating single-cell and spatial transcriptomics to elucidate intercellular tissue dynamics. Nat. Rev. Genet..

[7] Efremova M., Vento-Tormo M., Teichmann S.A., Vento-Tormo R. (2020). CellPhoneDB: Inferring cell-cell communication from combined expression of multi-subunit ligand-receptor complexes. Nat. Protoc..

[8] Chen J.G., Chávez-Fuentes J.C., O’Brien M., Xu J., Ruiz E., Wang W., Amin I., Sarfraz I., Guckhool P., Sistig A. (2023). Giotto Suite: A multi-scale and technology-agnostic spatial multi-omics analysis ecosystem. arXiv.

[9] Jin S., Guerrero-Juarez C.F., Zhang L., Chang I., Ramos R., Kuan C.H., Myung P., Plikus M.V., Nie Q. (2021). Inference and analysis of cell-cell communication using CellChat. Nat. Commun..

[10] Cheng C., Chen W., Jin H., Chen X. (2023). A Review of Single-Cell RNA-Seq Annotation, Integration, and Cell-Cell Communication. Cells.

[11] Yu D., Zhang S., Liu Z., Xu L., Chen L., Xie L. (2023). Single-Cell RNA Sequencing Analysis of Gene Regulatory Network Changes in the Development of Lung Adenocarcinoma. Biomolecules.

[12] Xu K., Yu D., Zhang S., Chen L., Liu Z., Xie L. (2024). Deciphering the Immune Microenvironment at the Forefront of Tumor Aggressiveness by Constructing a Regulatory Network with Single-Cell and Spatial Transcriptomic Data. Genes.

[13] Armingol E., Baghdassarian H.M., Lewis N.E. (2024). The diversification of methods for studying cell-cell interactions and communication. Nat. Rev. Genet..

[14] Peng L., Wang F., Wang Z., Tan J., Huang L., Tian X., Liu G., Zhou L. (2022). Cell-cell communication inference and analysis in the tumour microenvironments from single-cell transcriptomics: Data resources and computational strategies. Brief Bioinform..

[15] Dimitrov D., Türei D., Garrido-Rodriguez M., Burmedi P.L., Nagai J.S., Boys C., Ramirez Flores R.O., Kim H., Szalai B., Costa I.G. (2022). Comparison of methods and resources for cell-cell communication inference from single-cell RNA-Seq data. Nat. Commun..

[16] Feng Y., Ma W., Zang Y., Guo Y., Li Y., Zhang Y., Dong X., Liu Y., Zhan X., Pan Z. (2024). Spatially organized tumor-stroma boundary determines the efficacy of immunotherapy in colorectal cancer patients. Nat. Commun..

[17] Buechler M.B., Fu W., Turley S.J. (2021). Fibroblast-macrophage reciprocal interactions in health, fibrosis, and cancer. Immunity.

[18] Zhao Z., Li T., Yuan Y., Zhu Y. (2023). What is new in cancer-associated fibroblast biomarkers?. Cell Commun. Signal..

[19] Xia J., Xie Z., Niu G., Lu Z., Wang Z., Xing Y., Ren J., Hu Z., Hong R., Cao Z. (2023). Single-cell landscape and clinical outcomes of infiltrating B cells in colorectal cancer. Immunology.

[20] Chen Z., Zhang G., Ren X., Yao Z., Zhou Q., Ren X., Chen S., Xu L., Sun K., Zeng Q. (2023). Cross-talk between Myeloid and B Cells Shapes the Distinct Microenvironments of Primary and Secondary Liver Cancer. Cancer Res..

[21] Mani D.R., Krug K., Zhang B., Satpathy S., Clauser K.R., Ding L., Ellis M., Gillette M.A., Carr S.A. (2022). Cancer proteogenomics: Current impact and future prospects. Nat. Rev. Cancer.

[22] Guo T., Steen J.A., Mann M. (2025). Mass-spectrometry-based proteomics: From single cells to clinical applications. Nature.

[23] Uhlén M., Fagerberg L., Hallström B.M., Lindskog C., Oksvold P., Mardinoglu A., Sivertsson Å., Kampf C., Sjöstedt E., Asplund A. (2015). Proteomics. Tissue-based map of the human proteome. Science.

[24] Guilliams M., Bonnardel J., Haest B., Vanderborght B., Wagner C., Remmerie A., Bujko A., Martens L., Thoné T., Browaeys R. (2022). Spatial proteogenomics reveals distinct and evolutionarily conserved hepatic macrophage niches. Cell.

[25] Vandereyken K., Sifrim A., Thienpont B., Voet T. (2023). Methods and applications for single-cell and spatial multi-omics. Nat. Rev. Genet..

[26] Zheng X., Song J., Yu C., Zhou Z., Liu X., Yu J., Xu G., Yang J., He X., Bai X. (2022). Single-cell transcriptomic profiling unravels the adenoma-initiation role of protein tyrosine kinases during colorectal tumorigenesis. Signal Transduct. Target. Ther..

[27] Hua Y., Ma X., Zhao X., Wei X., Mu X., Zhang X. (2024). Characterization of metastasis-specific macrophages in colorectal cancer for prognosis prediction and immunometabolic remodeling. Sci. Rep..

[28] Du J., Zhang J., Wang L., Wang X., Zhao Y., Lu J., Fan T., Niu M., Zhang J., Cheng F. (2023). Selective oxidative protection leads to tissue topological changes orchestrated by macrophage during ulcerative colitis. Nat. Commun..

[29] Zhang J., Zhang M., Lou J., Wu L., Zhang S., Liu X., Ke Y., Zhao S., Song Z., Bai X. (2024). Machine Learning Integration with Single-Cell Transcriptome Sequencing Datasets Reveals the Impact of Tumor-Associated Neutrophils on the Immune Microenvironment and Immunotherapy Outcomes in Gastric Cancer. Int. J. Mol. Sci..

[30] Wu Y., Yang S., Ma J., Chen Z., Song G., Rao D., Cheng Y., Huang S., Liu Y., Jiang S. (2022). Spatiotemporal Immune Landscape of Colorectal Cancer Liver Metastasis at Single-Cell Level. Cancer Discov..

[31] Chen Y., Pal B., Lindeman G.J., Visvader J.E., Smyth G.K. (2022). R code and downstream analysis objects for the scRNA-seq atlas of normal and tumorigenic human breast tissue. Sci. Data.

[32] Parsons A., Colon E.S., Spasic M., Kurt B.B., Swarbrick A., Freedman R.A., Mittendorf E.A., van Galen P., McAllister S.S. (2024). Cell Populations in Human Breast Cancers are Molecularly and Biologically Distinct with Age. arXiv.

[33] Xu J., Fang Y., Chen K., Li S., Tang S., Ren Y., Cen Y., Fei W., Zhang B., Shen Y. (2022). Single-Cell RNA Sequencing Reveals the Tissue Architecture in Human High-Grade Serous Ovarian Cancer. Clin. Cancer Res..

[34] Zhang J., Liu X., Huang Z., Wu C., Zhang F., Han A., Stalin A., Lu S., Guo S., Huang J. (2023). T cell-related prognostic risk model and tumor immune environment modulation in lung adenocarcinoma based on single-cell and bulk RNA sequencing. Comput. Biol. Med..

[35] Butler A., Hoffman P., Smibert P., Papalexi E., Satija R. (2018). Integrating single-cell transcriptomic data across different conditions, technologies, and species. Nat. Biotechnol..

[36] Korsunsky I., Millard N., Fan J., Slowikowski K., Zhang F., Wei K., Baglaenko Y., Brenner M., Loh P.R., Raychaudhuri S. (2019). Fast, sensitive and accurate integration of single-cell data with Harmony. Nat. Methods.

[37] Aran D., Looney A.P., Liu L., Wu E., Fong V., Hsu A., Chak S., Naikawadi R.P., Wolters P.J., Abate A.R. (2019). Reference-based analysis of lung single-cell sequencing reveals a transitional profibrotic macrophage. Nat. Immunol..

[38] Hu C., Li T., Xu Y., Zhang X., Li F., Bai J., Chen J., Jiang W., Yang K., Ou Q. (2023). CellMarker 2.0: An updated database of manually curated cell markers in human/mouse and web tools based on scRNA-seq data. Nucleic Acids Res..

[39] Jin S., Plikus M.V., Nie Q. (2025). CellChat for systematic analysis of cell-cell communication from single-cell transcriptomics. Nat. Protoc..

[40] Xun Z., Ding X., Zhang Y., Zhang B., Lai S., Zou D., Zheng J., Chen G., Su B., Han L. (2023). Reconstruction of the tumor spatial microenvironment along the malignant-boundary-nonmalignant axis. Nat. Commun..

[41] Shao X., Li C., Yang H., Lu X., Liao J., Qian J., Wang K., Cheng J., Yang P., Chen H. (2022). Knowledge-graph-based cell-cell communication inference for spatially resolved transcriptomic data with SpaTalk. Nat. Commun..

[42] Pham D., Tan X., Balderson B., Xu J., Grice L.F., Yoon S., Willis E.F., Tran M., Lam P.Y., Raghubar A. (2023). Robust mapping of spatiotemporal trajectories and cell-cell interactions in healthy and diseased tissues. Nat. Commun..

[43] Van de Sande B., Flerin C., Davie K., De Waegeneer M., Hulselmans G., Aibar S., Seurinck R., Saelens W., Cannoodt R., Rouchon Q. (2020). A scalable SCENIC workflow for single-cell gene regulatory network analysis. Nat. Protoc..

[44] Xie L., Kong Q., Ai M., He A., Yao B., Zhang L., Zhang K., Zhu C., Li Y., Xia L. (2024). Spatial Proteomic Profiling of Colorectal Cancer Revealed Its Tumor Microenvironment Heterogeneity. J. Proteome. Res..

[45] Tang Z., Kang B., Li C., Chen T., Zhang Z. (2019). GEPIA2: An enhanced web server for large-scale expression profiling and interactive analysis. Nucleic Acids Res..

[46] Győrffy B. (2024). Integrated analysis of public datasets for the discovery and validation of survival-associated genes in solid tumors. Innovation.

[47] Walsh L.A., Quail D.F. (2023). Decoding the tumor microenvironment with spatial technologies. Nat. Immunol..

[48] Sternberg C., Raigel M., Limberger T., Trachtová K., Schlederer M., Lindner D., Kodajova P., Yang J., Ziegler R., Kalla J. (2024). Cell-autonomous IL6ST activation suppresses prostate cancer development via STAT3/ARF/p53-driven senescence and confers an immune-active tumor microenvironment. Mol. Cancer.

[49] Jones S.A., Jenkins B.J. (2018). Recent insights into targeting the IL-6 cytokine family in inflammatory diseases and cancer. Nat. Rev. Immunol..

[50] Kim D.H., Sung M., Park M.S., Sun E.G., Yoon S., Yoo K.H., Radhakrishnan K., Jung S.Y., Bae W.K., Cho S.H. (2024). Galectin 3-binding protein (LGALS3BP) depletion attenuates hepatic fibrosis by reducing transforming growth factor-β1 (TGF-β1) availability and inhibits hepatocarcinogenesis. Cancer Commun..

[51] Hou C., Wang D., Zhao M., Ballar P., Zhang X., Mei Q., Wang W., Li X., Sheng Q., Liu J. (2023). MANF brakes TLR4 signaling by competitively binding S100A8 with S100A9 to regulate macrophage phenotypes in hepatic fibrosis. Acta Pharm. Sin. B.

[52] Torretta S., Scagliola A., Ricci L., Mainini F., Di Marco S., Cuccovillo I., Kajaste-Rudnitski A., Sumpton D., Ryan K.M., Cardaci S. (2020). D-mannose suppresses macrophage IL-1β production. Nat. Commun..

[53] Tiwari A., Tashiro K., Dixit A., Soni A., Vogel K., Hall B., Shafqat I., Slaughter J., Param N., Le A. (2020). Loss of HIF1A From Pancreatic Cancer Cells Increases Expression of PPP1R1B and Degradation of p53 to Promote Invasion and Metastasis. Gastroenterology.

[54] Pérez-Gutiérrez L., Ferrara N. (2023). Biology and therapeutic targeting of vascular endothelial growth factor A. Nat. Rev. Mol. Cell. Biol..

[55] Lee C., Chen R., Sun G., Liu X., Lin X., He C., Xing L., Liu L., Jensen L.D., Kumar A. (2023). VEGF-B prevents excessive angiogenesis by inhibiting FGF2/FGFR1 pathway. Signal Transduct. Target. Ther..

[56] Ebrahimnezhad M., Valizadeh A., Majidinia M., Tabnak P., Yousefi B. (2024). Unveiling the potential of FOXO3 in lung cancer: From molecular insights to therapeutic prospects. Biomed. Pharmacother..

[57] Tsao A.S., Wei W., Kuhn E., Spencer L., Solis L.M., Suraokar M., Lee J.J., Hong W.K., Wistuba I.I. (2011). Immunohistochemical overexpression of platelet-derived growth factor receptor-beta (PDGFR-β) is associated with PDGFRB gene copy number gain in sarcomatoid non-small-cell lung cancer. Clin. Lung Cancer.

[58] Qian B.Z., Li J., Zhang H., Kitamura T., Zhang J., Campion L.R., Kaiser E.A., Snyder L.A., Pollard J.W. (2011). CCL2 recruits inflammatory monocytes to facilitate breast-tumour metastasis. Nature.

[59] Li J., Shu X., Xu J., Su S.M., Chan U.I., Mo L., Liu J., Zhang X., Adhav R., Chen Q. (2022). S100A9-CXCL12 activation in BRCA1-mutant breast cancer promotes an immunosuppressive microenvironment associated with resistance to immunotherapy. Nat. Commun..

[60] Davidson B., Stavnes H.T., Holth A., Chen X., Yang Y., Shih Ie M., Wang T.L. (2011). Gene expression signatures differentiate ovarian/peritoneal serous carcinoma from breast carcinoma in effusions. J. Cell. Mol. Med..

[61] Tian X., Song J., Zhang X., Yan M., Wang S., Wang Y., Xu L., Zhao L., Wei J.J., Shao C. (2020). MYC-regulated pseudogene HMGA1P6 promotes ovarian cancer malignancy via augmenting the oncogenic HMGA1/2. Cell Death Dis..

[62] Wang X., Tao X., Chen P., Jiang P., Li W., Chang H., Wei C., Lai X., Zhang H., Pan Y. (2024). MEK inhibition prevents CAR-T cell exhaustion and differentiation via downregulation of c-Fos and JunB. Signal Transduct. Target. Ther..

[63] Zhuang H., Zhou Z., Ma Z., Li Z., Liu C., Huang S., Zhang C., Hou B. (2020). Characterization of the prognostic and oncologic values of ITGB superfamily members in pancreatic cancer. J. Cell. Mol. Med..

[64] Wang X., Almet A.A., Nie Q. (2023). The promising application of cell-cell interaction analysis in cancer from single-cell and spatial transcriptomics. Semin. Cancer Biol..

[65] Mao X., Xu J., Wang W., Liang C., Hua J., Liu J., Zhang B., Meng Q., Yu X., Shi S. (2021). Crosstalk between cancer-associated fibroblasts and immune cells in the tumor microenvironment: New findings and future perspectives. Mol. Cancer.

[66] Zhang Y., Yu B., Ming W., Zhou X., Wang J., Chen D. (2024). SpaTopic: A statistical learning framework for exploring tumor spatial architecture from spatially resolved transcriptomic data. Sci. Adv..

