# Supplementary figures and images for: Construction of Gene Regulatory Networks Based on Spatial Multi-Omics Data and Application in Tumor-Boundary Analysis
Source: Genes (Basel). 2025 Jul 13;16(7):821. doi: 10.3390/genes16070821 (PMC12295195; doi:10.3390/genes16070821)

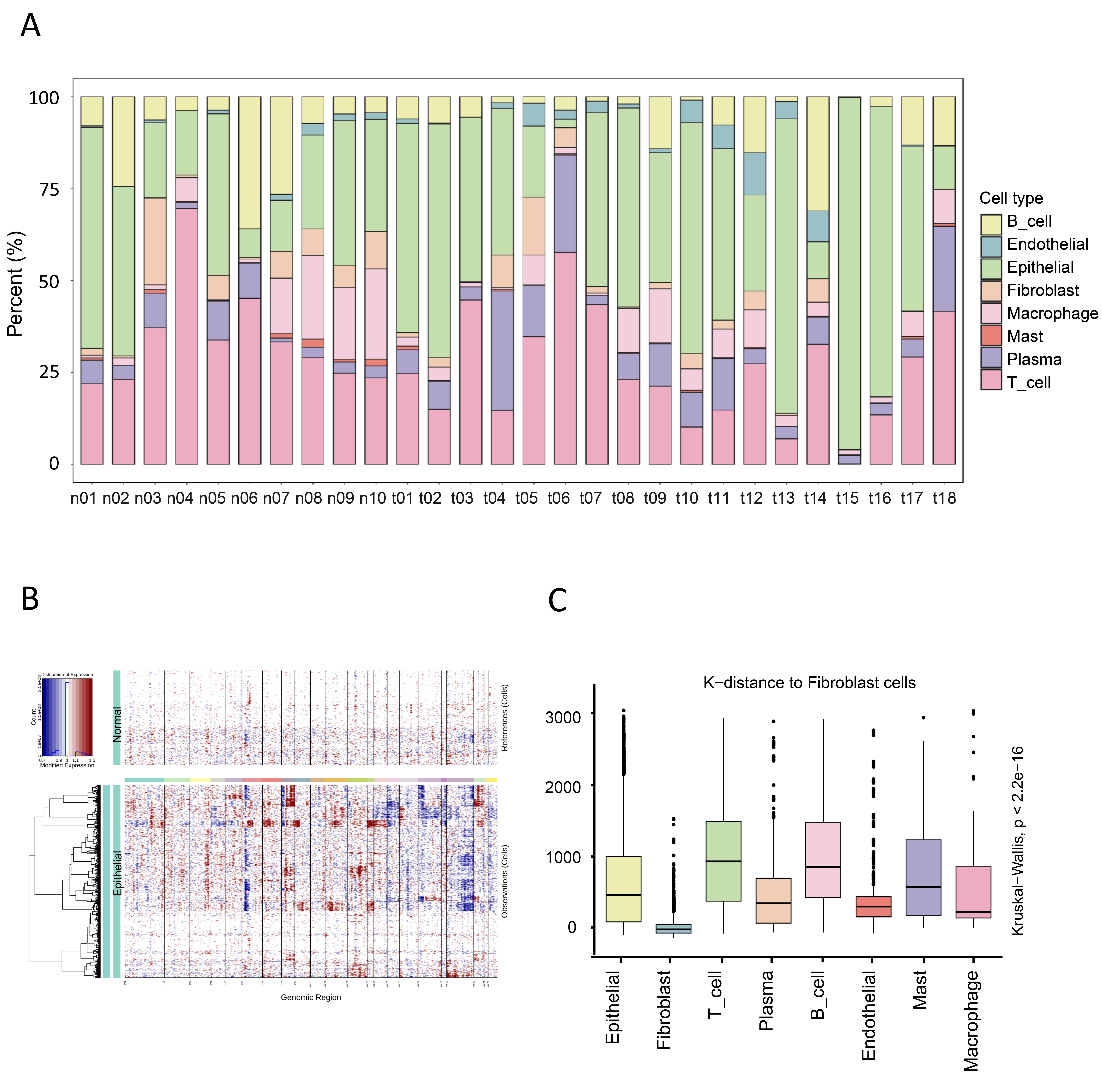

Supplement: Supplementary file 1 [file genes-16-00821-s001.zip › FigureS1.png]

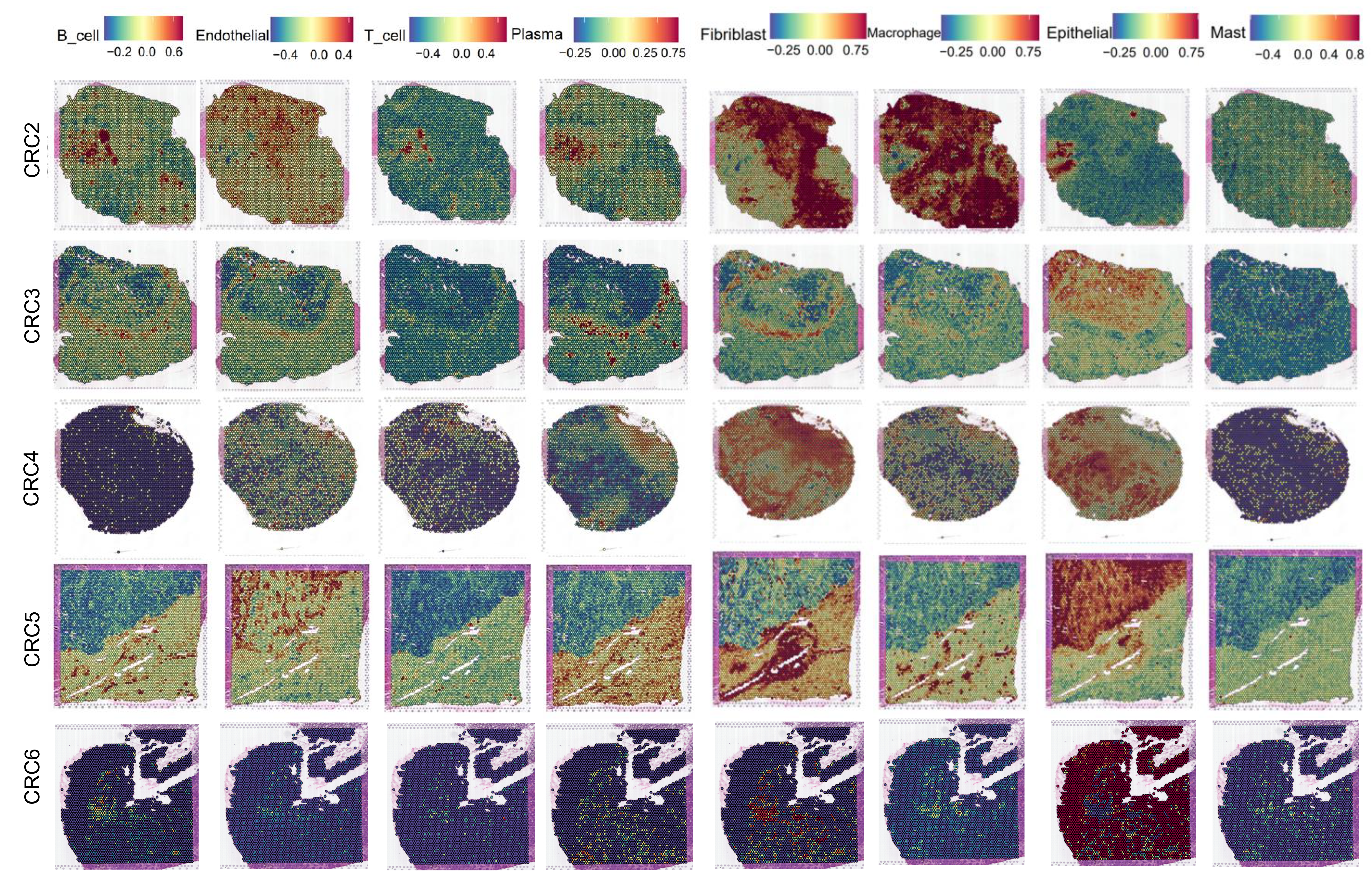

Supplement: Supplementary file 1 [file genes-16-00821-s001.zip › FigureS2.png]

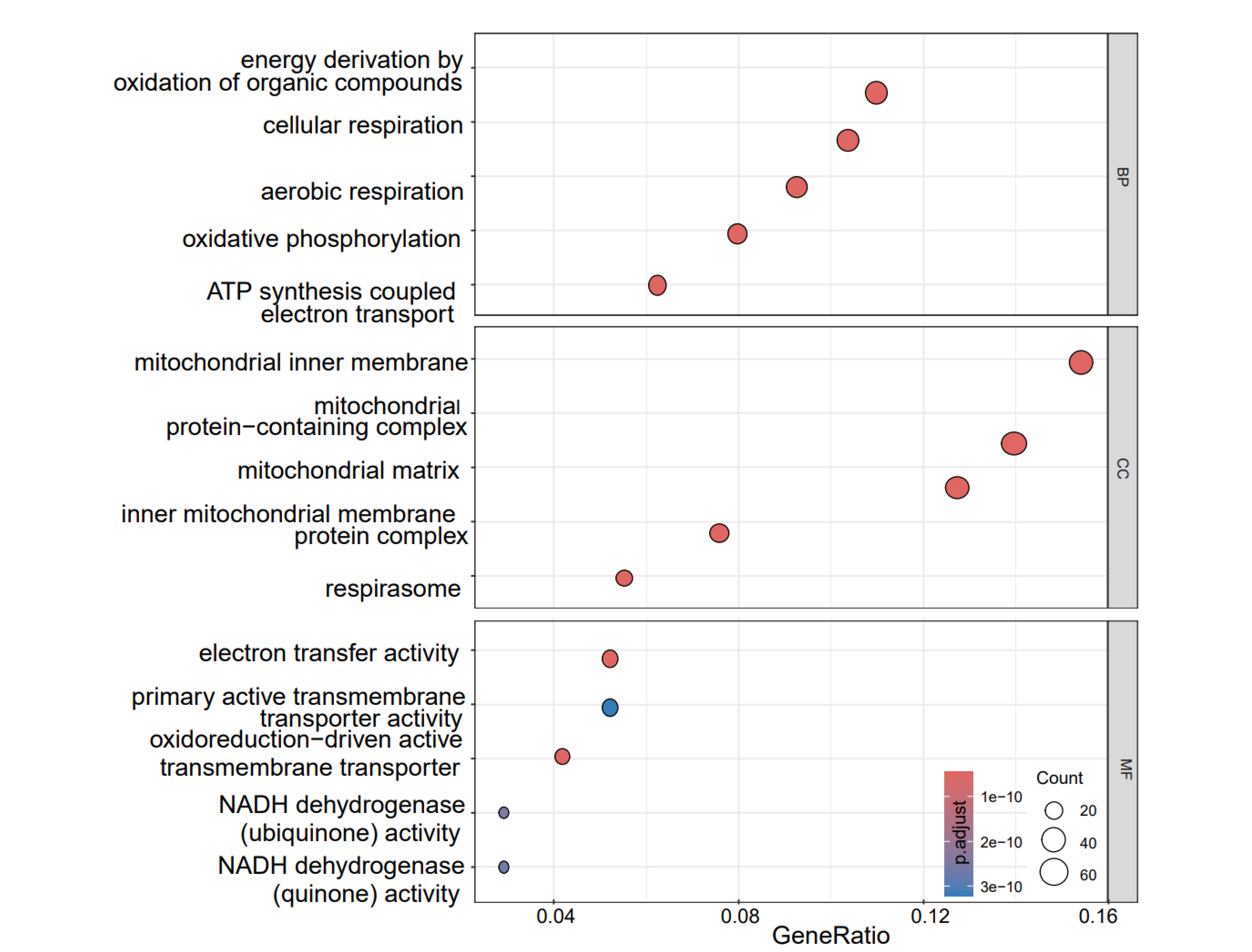

Supplement: Supplementary file 1 [file genes-16-00821-s001.zip › FigureS3.png]

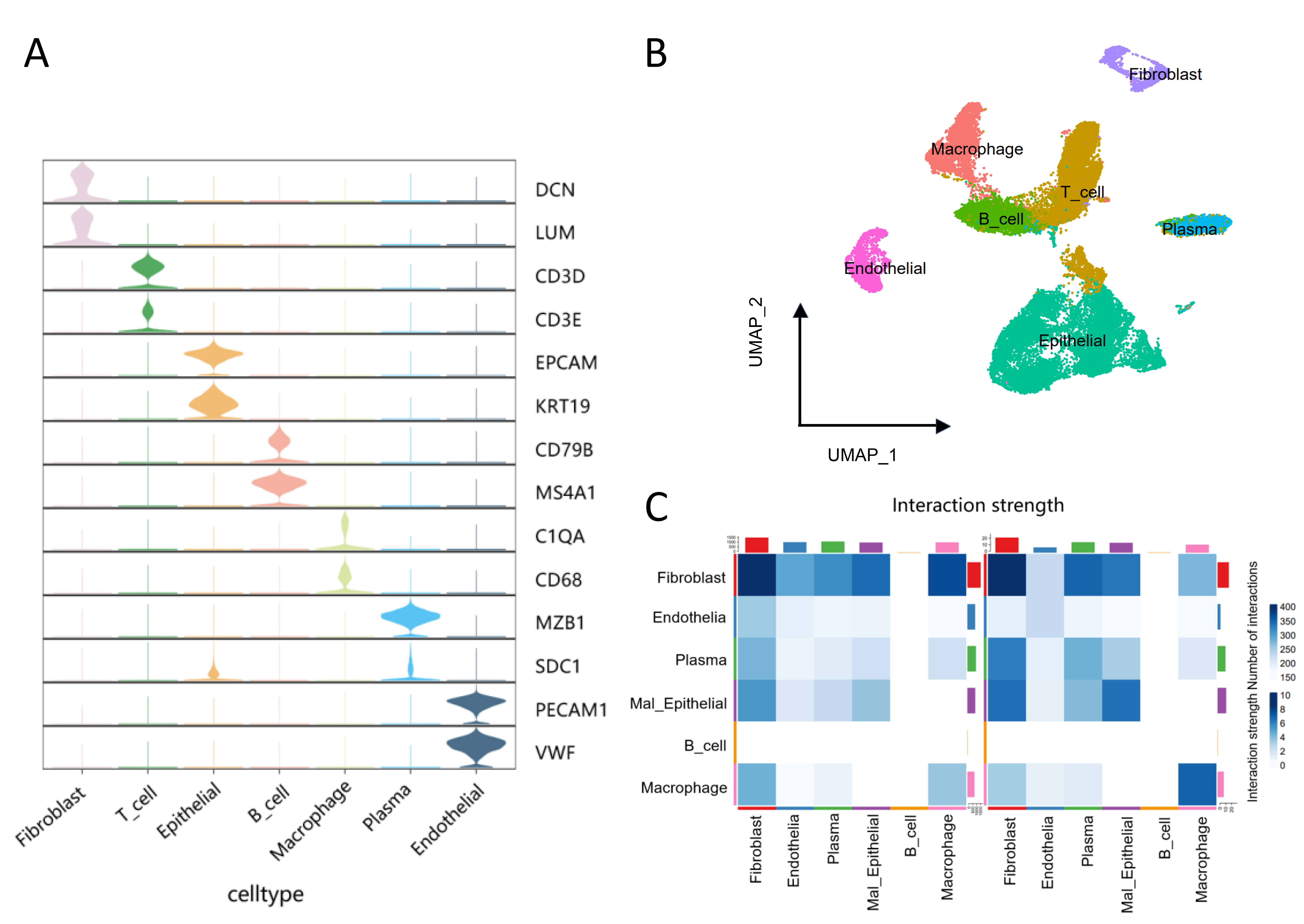

Supplement: Supplementary file 1 [file genes-16-00821-s001.zip › FigureS4.png]

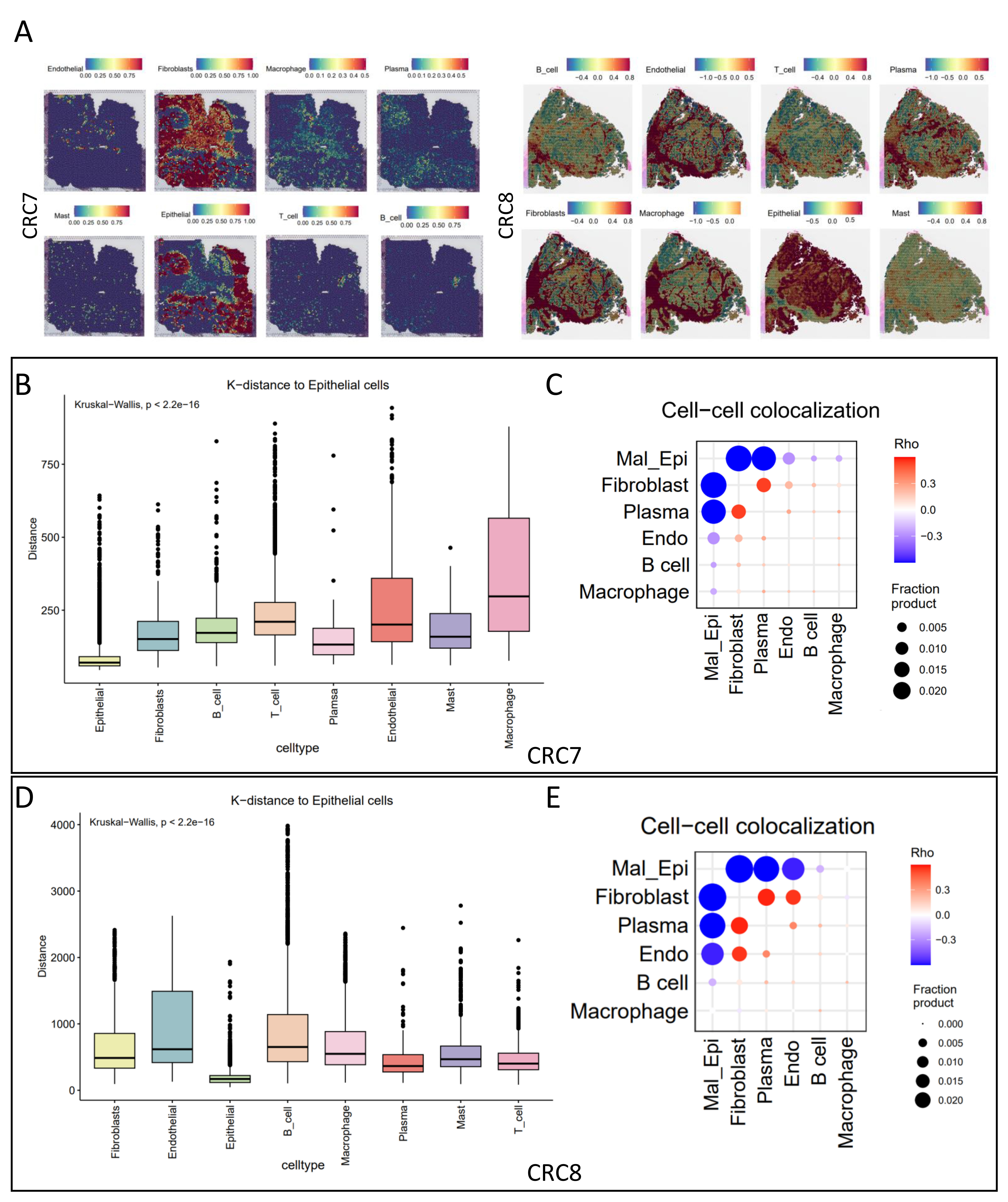

Supplement: Supplementary file 1 [file genes-16-00821-s001.zip › FigureS5.png]

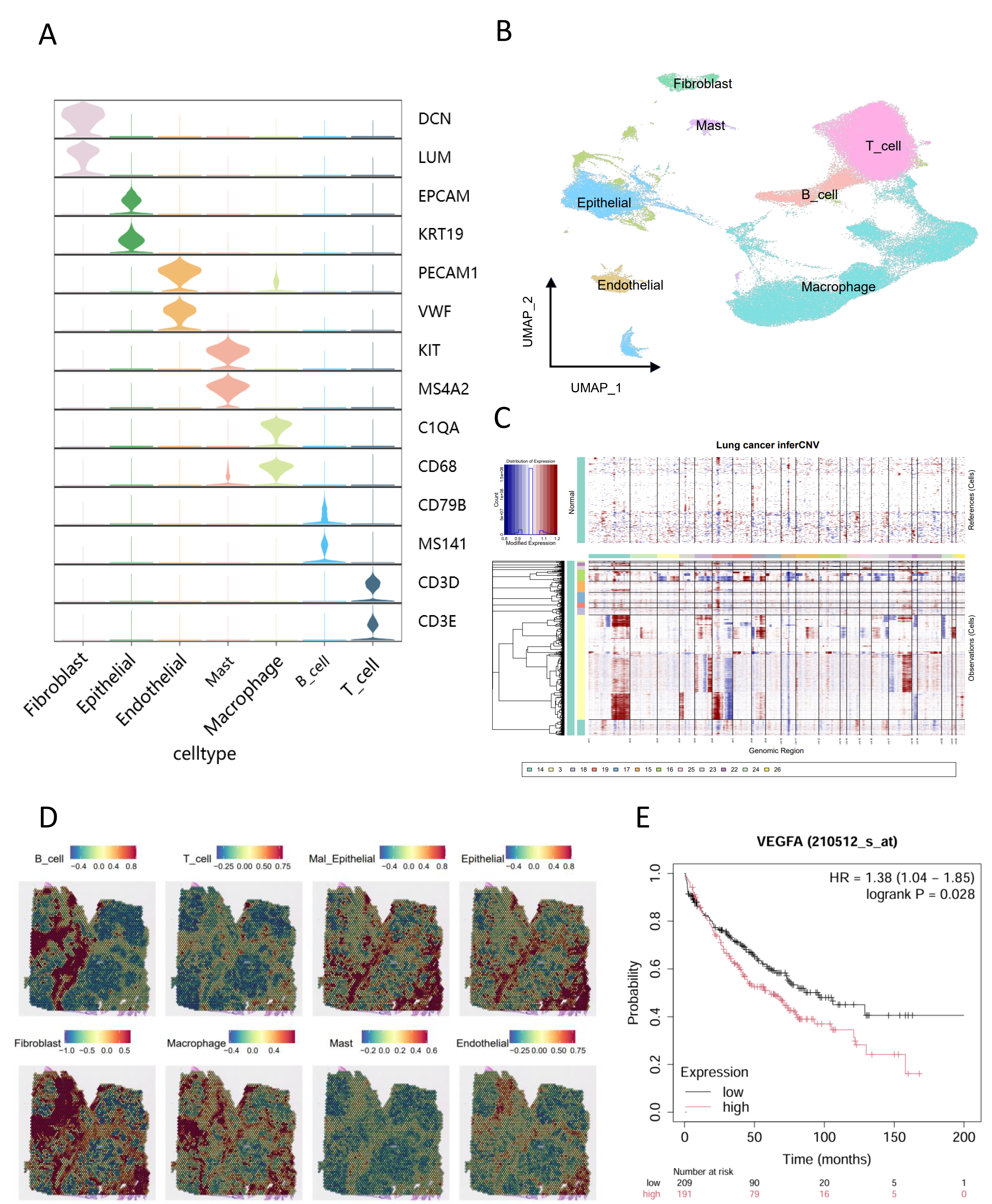

Supplement: Supplementary file 1 [file genes-16-00821-s001.zip › FigureS6.png]

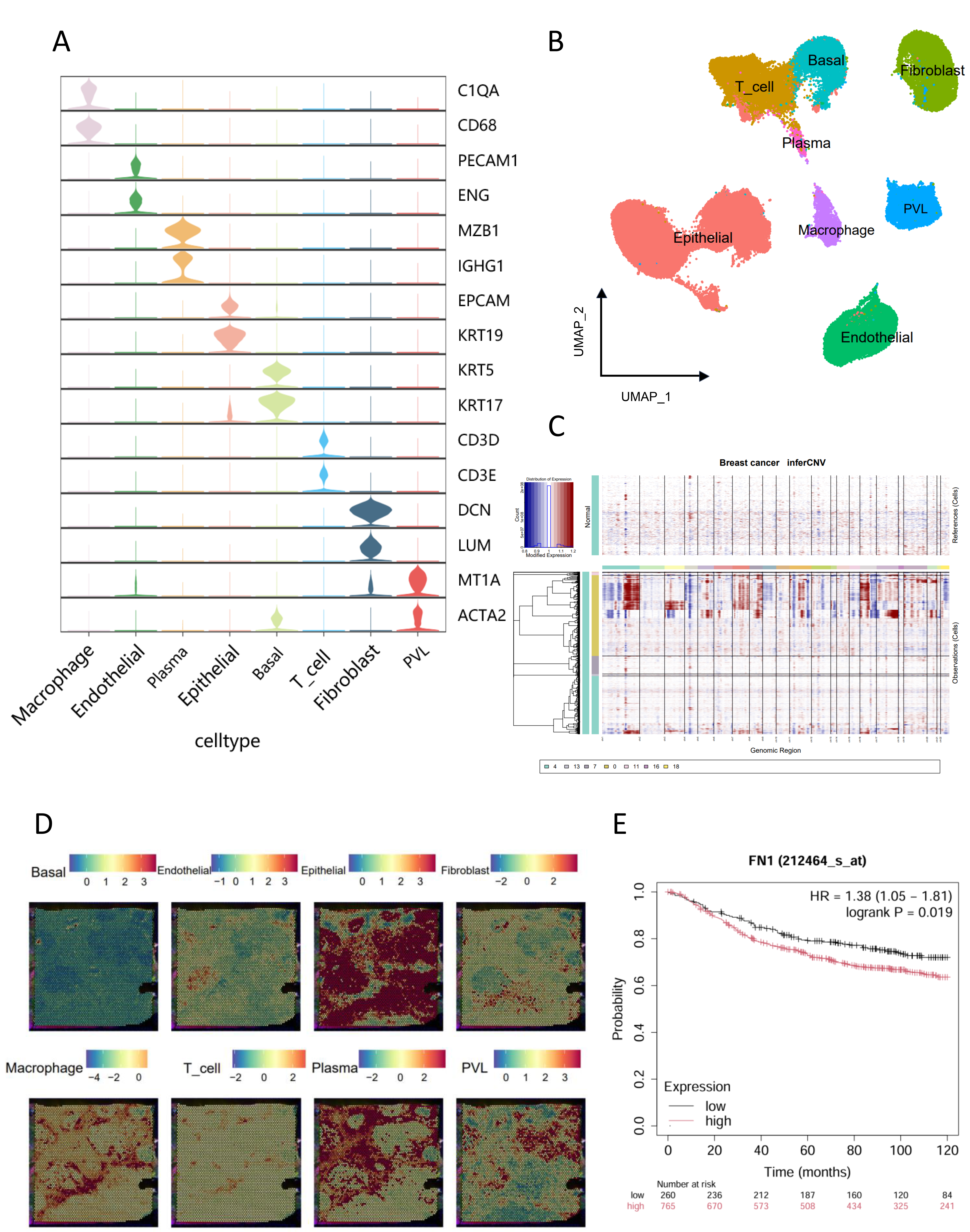

Supplement: Supplementary file 1 [file genes-16-00821-s001.zip › FigureS7.png]

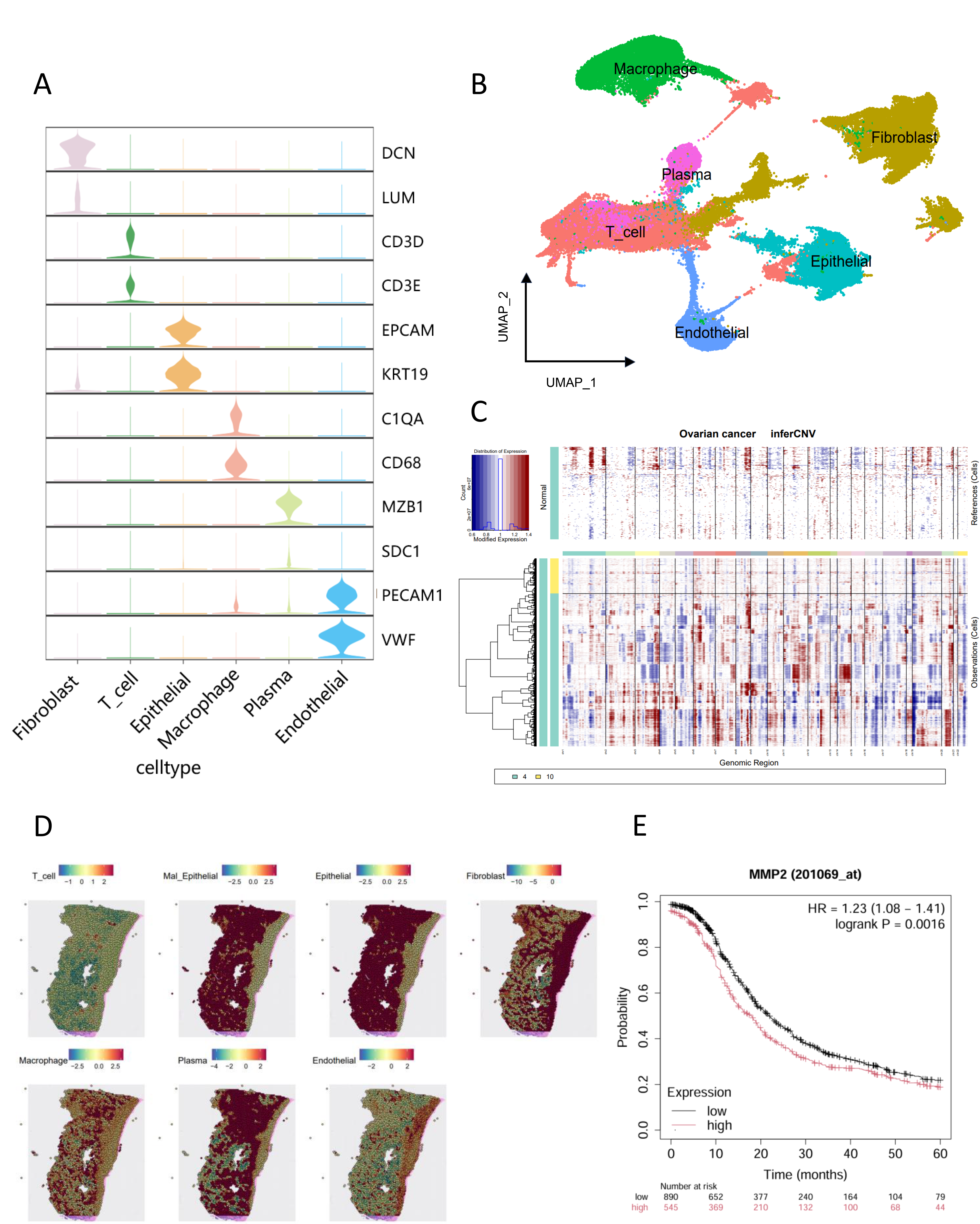

Supplement: Supplementary file 1 [file genes-16-00821-s001.zip › FigureS8.png]

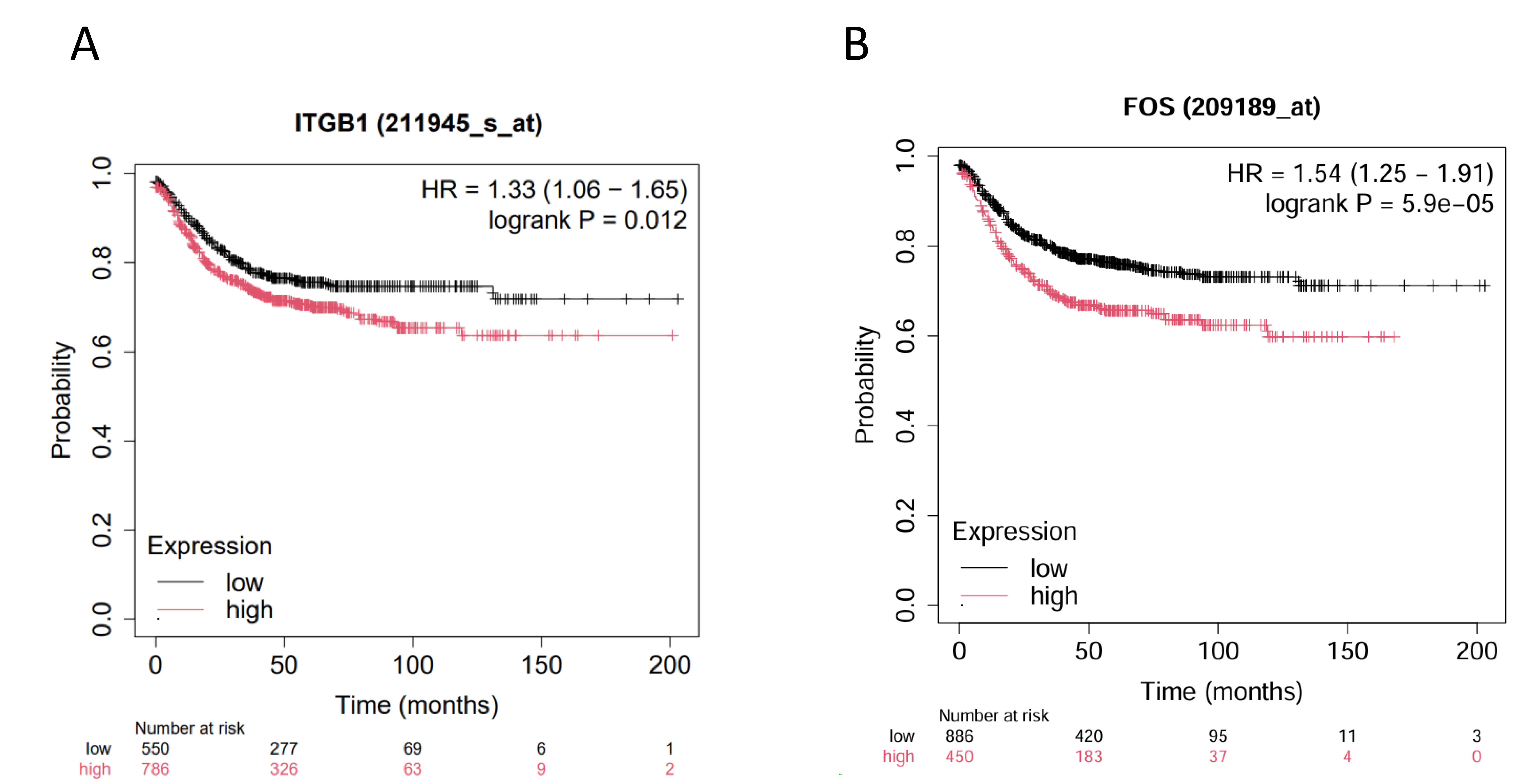

Supplement: Supplementary file 1 [file genes-16-00821-s001.zip › FigureS9.png]
